# Long Non-coding RNA FER1L4 Mediates the Autophagy of Periodontal Ligament Stem Cells Under Orthodontic Compressive Force via AKT/FOXO3 Pathway

**DOI:** 10.3389/fcell.2021.631181

**Published:** 2021-02-02

**Authors:** Yiping Huang, Hao Liu, Runzhi Guo, Yineng Han, Yuhui Yang, Yi Zhao, Yunfei Zheng, Lingfei Jia, Weiran Li

**Affiliations:** ^1^Department of Orthodontics, Peking University School and Hospital of Stomatology, Beijing, China; ^2^Central Laboratory, Peking University School and Hospital of Stomatology, Beijing, China; ^3^National Engineering Laboratory for Digital and Material Technology of Stomatology, Beijing Key Laboratory of Digital Stomatology, Beijing, China

**Keywords:** autophagy, FER1L4, long non-coding RNA, periodontal ligament stem cells, compressive force, orthodontic tooth movement

## Abstract

Orthodontic tooth movement is achieved by periodontal tissue remodeling triggered by mechanical force. It is essential to investigate the reaction of periodontal ligament stem cells (PDLSCs) for improving orthodontic therapeutic approaches. Autophagy is an endogenous defense mechanism to prevent mechanical damage of environmental change. Long non-coding RNAs (lncRNAs) are key regulators in gene regulation, but their roles are still largely uncharacterized in the reaction of PDLSCs during orthodontic tooth movement. In this study, we showed that autophagy was significantly induced in PDLSCs under compressive force, as revealed by the markers of autophagy, microtubule-associated protein light chain 3 (LC3) II/I and Beclin1, and the formation of autophagosomes. After the application of compressive force, lncRNA FER1L4 was strongly upregulated. Overexpression of FER1L4 increased the formation of autophagosome and autolysosomes in PDLSCs, while knockdown of FER1L4 reversed the autophagic activity induced by mechanical force. In mechanism, FER1L4 inhibited the phosphorylation of protein kinase B (AKT) and subsequently increased the nuclear translocation of forkhead box O3 (FOXO3) and thus mediated autophagic cascades under compressive strain. In mouse model, the expression of Lc3 as well as Fer1l4 was increased in the pressure side of periodontal ligament during tooth movement. These findings suggest a novel mechanism of autophagy regulation by lncRNA during periodontal tissue remodeling of orthodontic treatment.

## Introduction

Orthodontic tooth movement is achieved by the application of mechanical force, followed by periodontal tissue remodeling and regeneration (Krishnan and Davidovitch, 2006). Orthodontic force transferred from the teeth to the surrounding bone is mediated by the periodontal ligament (Krishnan and Davidovitch, 2009), which is a flexible connective tissue that attaches the root cementum to the alveolar bone (Beertsen et al., 1997). It induces bone deposition on the tension side and bone resorption on the compressive side when orthodontic force applied (Krishnan and Davidovitch, 2006, 2009). However, on the pressure side, necrosis or degeneration (hyalinization) of the periodontal ligament has been implicated in the application of heavy force (Hatai et al., 2001), while the reaction is more physiologic upon light force. Periodontal ligament stem cells (PDLSCs) are mechanosensitive cells that mediate the orthodontic force transduction and periodontal tissue remodeling (Jiang et al., 2016). Further investigation of the response of PDLSCs to the orthodontic force and a better understanding of this mechanism are essential to improve orthodontic therapeutic approaches.

Autophagy is a basic intracellular process mediating organelle degradation and recycling to maintain cellular homeostasis (Mizushima and Levine, 2010). In most cells, autophagy occurs at low level under normal condition, but is activated to survive in harsh environment, as a protective response (Tan et al., 2017). Previous studies have showed that autophagy serves as mechanical adaption in specialized mechanosensitive cells. It is increased in nucleus pulposus cells (Ma et al., 2013) in response to compressive force stimulus but decreased in podocytes (Li et al., 2016). However, it remains largely unknown about the role of autophagy in periodontal ligament under orthodontic compressive force. Recent study preliminarily found that autophagy is increased in periodontal ligament fibroblasts under biomechanical loading (Chen et al., 2019; Memmert et al., 2019). However, the precise mechanism by which the autophagy is regulated at the compressed area during tooth movement remains unclear.

Non-coding RNAs (ncRNAs) correspond to approximately 98% of the entire genome, playing essential regulatory roles in both physiological and pathophysiological control (Mattick, 2004). Long non-coding RNAs (lncRNAs) are a class of ncRNAs with transcript length > 200 nucleotides, emerging as key regulators in the field of autophagy (Yang et al., 2017). Recent reports suggest that lncRNAs involve in various cellular pathways by regulating autophagy, including ischemia and reperfusion injury (Tong et al., 2019; Yu et al., 2019), tumorigenesis, cardiovascular disease (Lorenzen and Thum, 2016; Viereck et al., 2016), and diabetes. In previous study, we have used RNA sequencing to detect the RNA landscape of PDLSCs exposed to static compressive force, and the results showed that 519 mRNAs and 90 lncRNAs were differentially expressed (Huang et al., 2019). Among these, Fer-1-like family member 4 (FER1L4) was one of the most highly upregulated transcripts. FER1L4 is a newly discovered lncRNA and has been showed to be related to some types of cancer, including lung carcinoma (Gao et al., 2019), hepatocellular cancer (Sun et al., 2019; Wang et al., 2019), osteosarcoma (Ye et al., 2019), and endometrial carcinoma (Qiao and Li, 2016). However, no study so far has investigated the function and mechanism of FER1L4 in autophagy. Thus, this research was aimed to determine the autophagic activity of PDLSCs under compressive force and further determine whether lncRNA FER1L4 is important for the autophagy of PDLSCs upon mechanical stress, and if so, what is the underlying mechanism.

## Materials and Methods

### Cell Isolation and Culture

This study was reviewed and approved by the Research Ethics Committee, Peking University School and Hospital of Stomatology (PKUSSIRB-2011007). Human PDLSCs were obtained from healthy premolars, which were extracted for orthodontic treatment. Briefly, the periodontal connective tissues were obtained from the middle of tooth roots, shred, and digested in a solution of collagenase type I and dispase for 30–60 min. Cell suspensions were filtered and cultured in α-modified Eagle’s medium (Gibco, Grand Island, NY, United States) supplemented with 10% fetal bovine serum (Gibco) and 100 units/mL pen/strep (Gibco). After 7-day incubation, cells were digested. They were further passaged and utilized until passage 4. The cell surface markers and multipotency were characterized as described previously (Zheng et al., 2017).

### Compressive Force Application

Periodontal ligament stem cells were seeded and cultured in 6-well plates. Upon 70–80% confluence, the cells were subjected to the static compressive force. A cover glass with additional metal balls was placed over the cell layer. The compressive force was set to 2.0 g/cm^2^ by adjusting the metal weights and maintained for 12 h according to previous study (Kanzaki et al., 2002).

### Plasmids

The FER1L4 expression plasmid was constructed using the pQLL vector by Qinglan Biotech Co. (Wuxi, China) and named pQLL-FER1L4. The pQLL vector was set as negative control (pQLL-NC).

### Oligoribonucleotides

The siRNAs against FER1L4 (siFER1L4) and the scrambled control (siNC) were designed by GenePharma Co. (Shanghai, China). The sequences of siFER1L4 are as follows: 5′-CAGGACAGCUUCGAGUUAATT-3′ (sense) and 5′-UUAACU CGAAGCUGUCCUGTT-3′ (antisense). The sequences of siNC are as follows: 5′-UUCUCCGAACGUGUCACGUTT-3′ (sense) and 5′-ACGUGACACGUUCGGAGAATT-3′ (antisense).

### Cell Transfection

Transfection of plasmids or siRNAs was performed using lipofectamine 3000 (Invitrogen, Carlsbad, CA, United States) according to the manufacturer’s protocols. Cells were seeded in 6-well plates. Once the cells reached 80–90% confluence, they were incubated in OPTI-MEM with Lipofectamine 3000 reagent and either plasmids (2 μg) or siRNAs (100 nM).

### Cell Proliferation Assay

Cell proliferation was determined using Cell Counting Kit-8 (CCK-8; Dojindo, Kumamoto, Japan) according to the manufacturer’s protocols. PDLSCs were exposed to the compressive stress for 12 h and cultured consecutively for 24, 48, and 72 h. The CCK-8 working solution was added and further incubated for 3 h. Absorbance was measured at 450 nm using a microplate reader (Bio-Rad, Hercules, CA, United States).

### Cell Apoptosis

Cell apoptosis was detected by Annexin V-FITC/PI double staining kit (Dojindo Laboratories, Kumamoto, Japan) according to the manufacturer’s instructions. Cells were washed and re-suspended in 1 ml binding buffer. Then 100 μl of the cell suspension were extracted and added to a new tube. Then, 5 μl Annexin V-FITC and 5 μl PI were added to each group. After incubation in the dark, 400 μl of binding buffer was added, and the cells were immediately detected by flow cytometry (BD LSR II, Becton Dickinson, Jersey, NJ, United States).

### Quantitative Real-Time Polymerase Chain Reaction (qRT-PCR)

Total RNA was extracted using TRIzol reagent (Invitrogen) following the manufacturer’s instructions. Then, 1 μg of total RNA was reverse-transcribed to cDNA using a PrimeScript^TM^ RT reagent Kit (Takara, Tokyo, Japan). The cDNA was amplified using SYBR Green PCR Master Mix (Applied Biosystems, Foster City, CA, United States) on an ABI 7500 Real-Time PCR System (Applied Biosystems). The housekeeping glyceraldehyde 3-phosphate dehydrogenase (GAPDH) gene was set as an internal control. The primers for FER1L4 are as follows: 5′-CCGTGTTGAGGTGCTGTTC-3′ (sense) and 5′-GGCAAGTCCACTGTCAGATG-3′ (antisense). The primers for GAPDH are as follows: 5′-GGTCACCAGGGCTGCTTTTA-3′ (sense) and 5′-GGATCTCGCTCCTGGAAGATG-3′ (antisense). The 2^–Δ^ ^Δ^ ^*Ct*^ method was used to analyze the relative gene expression as described previously (Huang et al., 2015).

### Western Blot

The immunoblotting assay was performed according to previous study (Huang et al., 2015). Briefly, cells were lysed in RIPA buffer with protease inhibitor cocktail (Solarbio, Beijing, China). The bicinchoninic acid method was used to determine the protein content. Proteins were separated by SDS-PAGE gels before electroblotted to PVDF membranes (Millipore, Billerica, MA, United States). The membranes were blocked in 5% fat-free milk for 1 h, followed by incubation with primary antibodies against protein kinase B (AKT; Abcam, Cambridge, UK), p-AKT (Abcam), forkhead box O3 (FOXO3; Abcam), p-FOXO3 (Abcam), Beclin1 (Abcam), microtubule-associated protein light chain 3 (LC3; Cell Signaling Technology, Beverly, MA, United States), and GAPDH (Zhongshan Goldenbridge Biotechnology Co., Beijing, China) at 4°C overnight. The next day, the membranes were washed before incubation with secondary antibodies (Zhongshan Goldenbridge Biotechnology Co.) for 1 h. The blots were detected using an enhanced chemiluminescence substrate kit (Applygen, Beijing, China) and quantified using ImageJ software. The intensity of each signal was normalized to GAPDH values.

### Immunofluorescence Analysis

Cells grown on glass coverslips were fixed in 4% paraformaldehyde at room temperature for 20 min, permeabilized with 0.1% Triton X-100 for 15 min, blocked in 10% goat serum (Zhongshan Goldenbridge Biotechnology Co.) for 1 h, and then incubated with primary antibodies against LC3 (Cell Signaling Technology) and FOXO3 (Abcam) at 4°C overnight. Subsequently, cells were incubated with fluorescent secondary antibody (Zhongshan Goldenbridge Biotechnology Co.) for 1 h. The cell nuclei were counterstained with the blue-fluorescent DNA stain DAPI (Zhongshan Goldenbridge Biotechnology Co.). Glass coverslips were mounted on a microscope slide and images were acquired using confocal laser-scanning microscope (Carl Zeiss, Jena, Germany).

### Fluorescence *in situ* Hybridization on Cells

The assay was performed using Ribo^TM^ fluorescence *in situ* hybridization (FISH) Kit (RiboBio Co., Guangzhou, China). Cells were cultured on glass coverslips, washed in PBS, immersed in 4% paraformaldehyde at room temperature for 30 min, treated with 0.5% Triton X-100 in PBS at 4°C for 5 min, and prehybridizated at 37°C for 30 min before hybridization. After that, the probe (RiboBio Co.) in the hybridization solution was applied to the cells at 37°C overnight. The slides were washed and mounted using DAPI for detection. The fluorescent images were captured using a confocal microscope (Carl Zeiss).

### Transmission Electron Microscope

Transmission electron microscope (TEM) assessment was performed according to previous study (Tang et al., 2011). In brief, cells were harvested and fixed in 2.5% glutaraldehyde, followed by incubation in 1% OsO4 at 4°C overnight. After stepwise dehydration in ascending alcohols, the cells were embedded in epoxy-araldite resin following standard protocols. Subsequently, sections were cut, mounted on copper grids, stained with uranyl acetate and lead citrate, and examined under a transmission electron microscope (FEI TECNAI, Hillsboro, OR, United States).

### Animal Experiment for Orthodontic Tooth Movement

The experimental protocol was reviewed and approved by Institutional Animal Care and Use Committee, Peking University (LA2018305). The male BALB/c mice (6–8 weeks, 20–25 g) were obtained from Vital River Laboratory Animal Technology Co. (Beijing, China). The application of orthodontic force was conducted as described previously (Wang et al., 2018). In brief, a nickel-titanium coil spring was inserted between the maxillary left first molar and maxillary incisors using flowable composite resin. The force was adjusted to 20 g. The maxillary right first molar was used as the control tooth. After 3 days, the maxilla were obtained and immersed in 4% paraformaldehyde.

### Micro-Computed Tomography Analysis

Images were acquired with uniform parameters using Inveon Micro-CT scanning (Siemens, Munich, Germany). Three-dimensional images were reconstructed using Inveon Research Workplace (Siemens).

### Hematoxylin and Eosin Staining

Each sample was decalcified in 10% EDTA at 4°C for 4 weeks. Then, the specimens were embedded in paraffin, and transverse sections of the first molar region were cut at 5 μm. Hematoxylin and eosin (H&E) staining was performed following standard protocols.

### Immunohistochemical Staining

Immunohistochemical staining was conducted as described previously (Huang et al., 2015). In brief, the tissue sections were deparaffined in xylene and rehydrated in graded alcohols. For antigen retrieval, citric acid antigen repair buffer was used for 15 min. After endogenous peroxidase activity was quenched with 3% H_2_O_2_ for 10 min, the sections were saturated with 10% goat serum for 30 min. The sections were then incubated with primary antibody against Lc3 (Cell Signaling Technology) at 4°C overnight. The following day, the slides were rinsed with PBS, incubated with secondary antibody (Zhongshan Goldenbridge Biotechnology Co.), followed by incubation in diaminobenzidine solution. The development of brown chromogen product was monitored under a microscope. Hematoxylin was used to dye the nucleus blue, and the tissue was dehydrated. The staining was visualized with the Olympus BX51 microscope, and images were captured with digital camera (Olympus Co., Tokyo, Japan).

### FISH on Tissue Sections

The FISH assay on tissue sections was conducted according to previous study (Larsen et al., 2018). In brief, the sections were deparaffined and rehydrated with a series of graded alcohols into distilled water. After pre-treatment, the slices were immersed into pre-warmed pepsin solution and then dehydrated in graded alcohols. The sections were hybridized in the Fer1l4 probe (Ribobio Co.) cocktail overnight. The slides were then washed, air-dried, and coverslipped with mounting media with DAPI. The probe signals were visualized under fluorescence microscopy (Carl Zeiss).

### Statistics

All data are expressed as the mean ± SD from at least three biologically repeated experiments with three technical replicates per sample. Differences between parametric data of two groups were assessed using the Student’s *t*-test. Differences among multiple groups were analyzed using one-way ANOVA followed by Tukey’s multiple-comparison test. Differences with *p*-value < 0.05 were considered statistically significant.

## Results

### Autophagy Is Induced in PDLSCs Under Compressive Force

**FIGURE 1:**
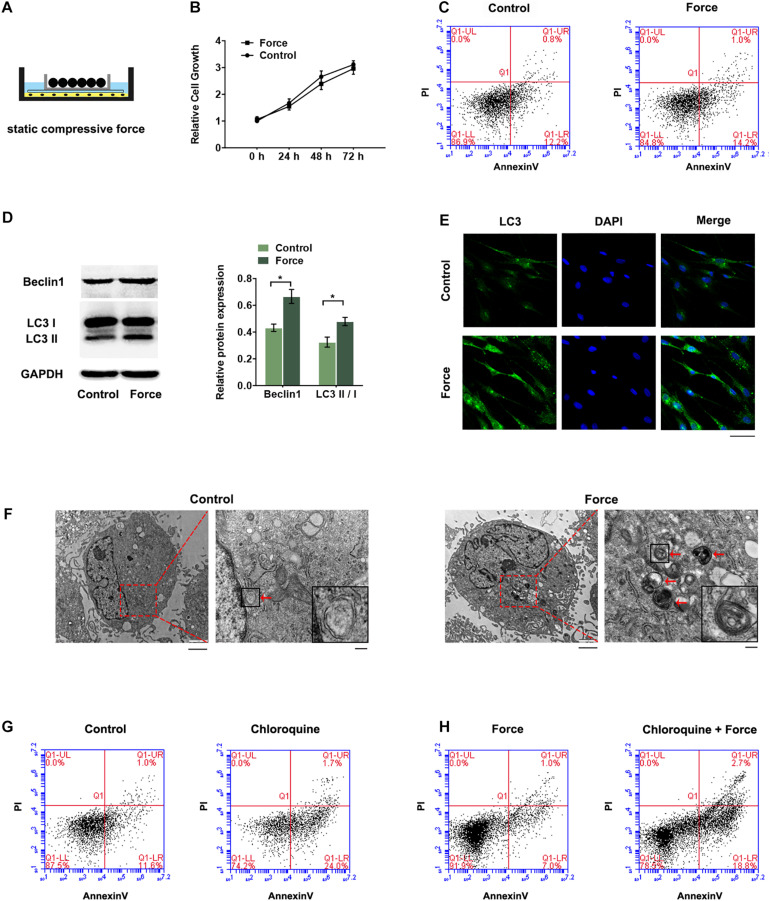
Autophagy was increased in PDLSCs under compressive force. Cells were subjected to static compressive force for 12 h. **(A)** Schematic diagram of compressive force applied. **(B)** Relative cell growth of PDLSCs with or without compressive force. **(C)** Apoptotic cells were monitored with FACS after Annexin V and PI staining. **(D)** Western blot analyses of Beclin1 and LC3II/LC3I. Histograms show the quantification of the band intensities. **(E)** Images of LC3 immunofluorescence staining. Scale bar: 50 μm. **(F)** Ultrastructural features assessed by electron microscopy in the control group and force group (arrows point to the autophagosomes or autolysosomes). Scale bar: 2 μm (left) and 500 nm (right). **(G)** Flow cytometry of Annexin V-FITC/PI staining in PDLSCs with or without chloroquine treatment (10 μM). **(H)** Flow cytometry of Annexin V-FITC/PI staining in PDLSCs under compressive force with or without chloroquine pre-treatment. Data are presented as the mean ± SD (*n* = 3, *p < 0.05).

Periodontal ligament stem cells were exposed to static compressive force for 12 h (Figure 1A). The CCK-8 assay showed that the cell proliferation was similar between the groups with or without compressive force (Figure 1B). The flow cytometry analyses also confirmed no significant difference of cell apoptosis and necrosis in the force group compared with the control group (Figure 1C). The autophagic activity was further determined. The expression levels of two biological markers of autophagy, LC3 II/I and Beclin1, were higher in the force group (Figure 1D). The presence of autophagosomes was detected as punctate dots under fluorescence microscopy. Consistently, the LC3 punctate dots were remarkable around the perinuclear and cytoplasm regions in the force group (Figure 1E). TEM was also used to detect autophagy flux. Autophagosomes, which are defined as double-membrane vesicles containing cellular debris or degrading organelles in the cytoplasm of PDLSCs, were increased in the compressive force group (Figure 1F). To determine the role of autophagy, we treated PDLSCs with chloroquine (10 μM), an autophagy inhibitor. The results showed that the apoptotic cells increased significantly after the inhibition of autophagy flux (Figure 1G). When the cells were pre-treated with the autophagy inhibitor, the light compressive force (2 g/cm^2^) induced significant cell apoptosis (Figure 1H).

### LncRNA FER1L4 Is Significantly Upregulated in PDLSCs Subjected to Compressive Force

**FIGURE 2:**
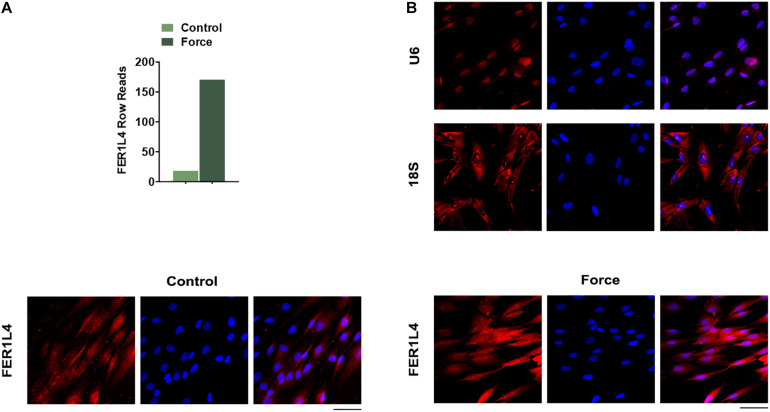
FER1L4 was upregulated after the application of compressive force in PDLSCs. **(A)** Relative expression of FER1L4 determined by RNA sequencing after 12 h compression of PDLSCs. **(B)** FISH images of U6, 18S, and FER1L4. Scale bar: 50 μm (*n* = 3).

Previous raw data of RNA sequencing showed that FER1L4 was strongly increased (∼7-fold) in PDLSCs subjected to compressive force (Figure 2A). To further study the role of FER1L4, the distribution of FER1L4 was visualized under fluorescent microscope using FISH technique. The results showed that FER1L4 was located in both the nucleus and the cytoplasm of the cells. After the static compressive force stimulation, the intensity of FER1L4 in both the nucleus and the cytoplasm was increased (Figure 2B).

### FER1L4 Mediates the Autophagy of PDLSCs Induced by the Compressive Force

**FIGURE 3:**
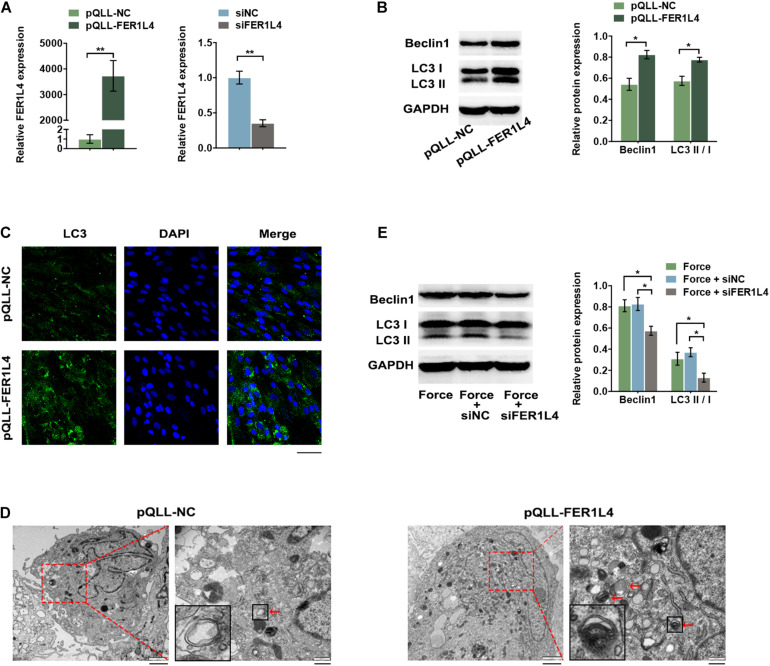
FER1L4 promoted the level of autophagy in PDLSCs. Cells were transfected with FER1L4 vector (pQLL-FER1L4) or FER1L4 siRNA (siFER1L4) to overexpress or knock down its expression. **(A)** Relative FER1L4 expression in the FER1L4 overexpression and knockdown groups. **(B)** Western blot analyses of Beclin1 and LC3II/LC3I. Histograms show the quantification of the band intensities. **(C)** Images of LC3 immunofluorescence staining. Scale bar: 50 μm. **(D)** Ultrastructural features assessed by electron microscopy in FER1L4 overexpression group and control group (arrows point to the autophagosomes or autolysosomes). Scale bar: 2 μm (left) and 500 nm (right). **(E)** Western blot analyses of Beclin1 and LC3II/LC3I after the application of compressive force with or without FER1L4 knockdown. Histograms show the quantification of the band intensities. Data are presented as the mean ± SD (*n* = 3, *p < 0.05; **p < 0.01).

To investigate the role of FER1L4 in regulation of autophagy in PDLSCs, gain-of-function and loss-of-function assays were performed. The expression of FER1L4 was markedly increased in pQLL-FER1L4 plasmid-transfected group and decreased significantly in siFER1L4-transfected group (Figure 3A).

After transfection with pQLL-FER1L4 vector, the protein expression of autophagic markers LC3 II/I and Beclin1 was upregulated as demonstrated by the western blot analysis (Figure 3B). The cytoplasmic formation of LC3 puncta was significantly increased in FER1L4 overexpression group under confocal microscope, indicating a significant increase of formation of autophagosomes (Figure 3C). The formation of autophagosome and autolysosomes was further confirmed by TEM. The number of autophagosome and autolysosomes in the cytoplasm of PDLSCs was higher after transfected with FER1L4 vector (Figure 3D).

To determine whether FER1L4 mediates the autophagy of PDLSCs under compressive force, the cells were pre-transfected with siFER1L4 and then exposed to mechanical stress for 12 h; the LC3 II/I and Beclin1 induced by mechanical stress were abolished by FER1L4 knockdown (Figure 3E).

### FER1L4 Regulates Autophagy via AKT/FOXO3 Signaling Pathway During the Application of External Compressive Force

**FIGURE 4:**
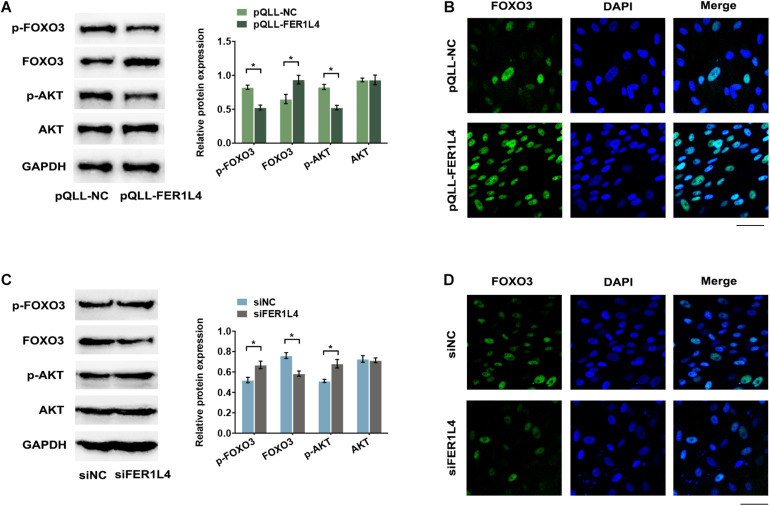
FER1L4 inhibited the phosphorylation of AKT and increased the nuclear translocation of FOXO3. Cells were transfected with FER1L4 vector (pQLL-FER1L4) or FER1L4 siRNA (siFER1L4) to overexpress or knock down its expression. **(A)** Western blot analyses of p-AKT, AKT, p-FOXO3, and FOXO3 in FER1L4 overexpression group. Histograms show the quantification of the band intensities. **(B)** Images of FOXO3 immunofluorescence staining in FER1L4 overexpression group. Scale bar: 50 μm. **(C)** Western blot analyses of p-AKT, AKT, p-FOXO3, and FOXO3 in FER1L4 knockdown group. Histograms show the quantification of the band intensities. **(D)** Images of FOXO3 immunofluorescence staining in FER1L4 knockdown group. Scale bar: 50 μm. Data are presented as the mean ± SD (*n* = 3, *p < 0.05).

AKT signaling pathway is important in negative regulation of autophagy. To investigate whether FER1L4 regulates AKT signaling pathway in PDLSCs, we performed western blot analysis and the result showed that FER1L4 overexpression inhibited the phosphorylation of AKT (p-AKT), while the total AKT remained almost unchanged (Figure 4A). Concomitant with the decrease of p-AKT, a significant decrease of p-FOXO3 and increase of FOXO3 expression were observed (Figure 4A). The localization of FOXO3 protein was also detected by immunofluorescence assay. Strong immunostaining of FOXO3 was noted in FER1L4 overexpressed group (Figure 4B). While, in FER1L4 knockdown cells, the phosphorylation of AKT and the phosphorylation of FOXO3 were upregulated, and the expression of FOXO3 was downregulated (Figure 4C). Immunostaining also showed relative weak fluorescent staining of FOXO3 in FER1L4 knockdown group (Figure 4D).

**FIGURE 5:**
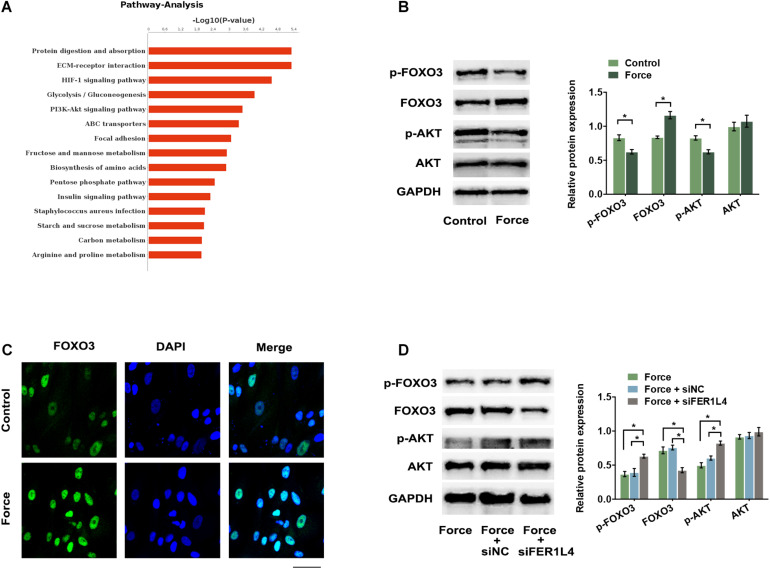
Compressive force regulated AKT/FOXO3 pathway and FER1L4 partially mediated this process. **(A)** The top 15 enrichments in the KEGG pathway analysis of differentially expressed genes in PDLSCs under compressive force. PI3K/AKT signaling pathway was significantly affected. **(B)** Western blot analyses of p-AKT, AKT, p-FOXO3, and FOXO3 in the force and control group. Histograms show the quantification of the band intensities. **(C)** Images of FOXO3 immunofluorescence staining in PDLSCs under compression. Scale bar: 50 μm. **(D)** Western blot analyses of p-AKT, AKT, p-FOXO3, and FOXO3 after the application of compressive force with or without FER1L4 knockdown. Histograms show the quantification of the band intensities. Data are presented as the mean ± SD (*n* = 3, *p < 0.05).

To further determine whether FER1L4 mediates the autophagy of compressed PDLSCs via AKT/FOXO3 signaling, we then investigated the activation of this pathway during the application of compressive force. Pathway analysis of previous RNA sequencing data based on Kyoto Encyclopedia of Genes and Genomes (KEGG) database showed that PI3K/AKT signaling pathway was significantly affected under mechanical stress (Figure 5A). Consistently, p-AKT and p-FOXO3 were significantly reduced (Figure 5B), while the nuclear translocation of FOXO3 increased in PDLSCs exposed to compressive force (Figure 5C). However, when the cells were pretransfected with siFER1L4, the inhibition of p-AKT and p-FOXO3 and the activation of FOXO3 induced by the compressive force were lost by FER1L4 knockdown (Figure 5D).

### Fer1l4 and Autophagy Marker Are Upregulated in Compressed Periodontal Ligament of Orthodontic Tooth

**FIGURE 6:**
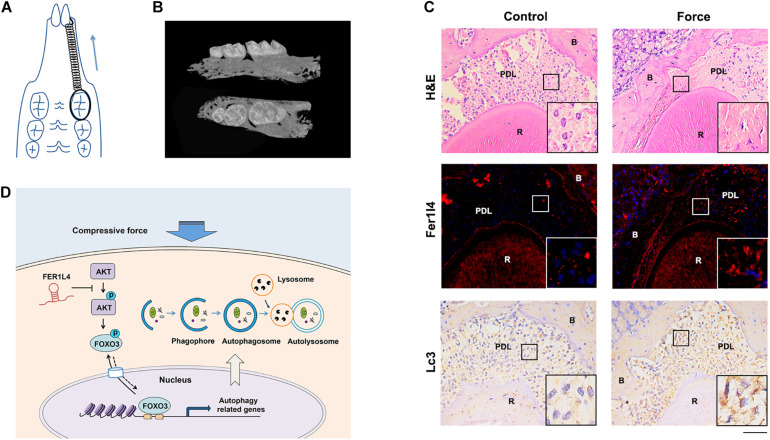
Fer1l4 and Lc3 were upregulated in compressed periodontal ligament during orthodontic tooth movement. **(A)** Schematic diagram of mouse orthodontic tooth movement model. **(B)** Reconstructed three-dimensional micro-CT images of tooth movement. **(C)** H&E staining, FISH images of Fer1l4, and immunohistochemical staining of Lc3 in periodontal ligament of upper first molar with or without compressive strain. Scale bar: 100 μm. (*n* = 3). **(D)** Schematic diagram showing FER1L4 mediates the autophagy of periodontal ligament stem cells under orthodontic compressive force via AKT/FOXO3 pathway.

To determine the function of FER1L4 and autophagy in reaction of periodontal ligament to the orthodontic force, we used a mouse orthodontic tooth movement model by bonding the maxillary left first molar to the incisors with nickel-titanium coil springs providing ∼20 g orthodontic force continuously (Figure 6A), and the right maxillary first molar was set as control tooth. Micro-CT data demonstrated the successful mesial movement of the first molar (Figure 6B). Histological examination showed that the periodontal ligament of pressure side was compressed by the experimental tooth against the alveolar bone. FISH assay revealed that the intensity of fluorescence staining of Fer1l4 RNA was significantly induced in the compressed areas of the periodontal ligament, while the fluorescence intensity was rather low in the control periodontal ligament (Figure 6C). The autophagy in the compressed periodontal ligament of orthodontic tooth was also determined. Immunohistochemical staining showed increased activity of autophagy marker Lc3 in the region where the orthodontic compression was applied (Figure 6C).

## Discussion

In this study, we showed an increase of autophagy in PDLSCs under compressive force. PDLSCs are force-sensitive cells that react to mechanical stimulation and mediate periodontal tissue remodeling during orthodontic treatment (Jiang et al., 2016). Previous studies have showed that autophagy is regulated in specialized mechanosensitive cells, such as nucleus pulposus cells (Ma et al., 2013), podocytes (Li et al., 2016), and skeletal muscle cells (Teng et al., 2011). In current study, autophagy was activated in PDLSCs in response to compressive stress, consistent with the recent preliminary study (Chen et al., 2019). Autophagy is a form of endogenous defense mechanism to maintain cellular homeostasis and prevent mechanical damage of environmental change (Mizushima and Levine, 2010). Under mechanical loading, the autophagy-dependent mechanism is activated to remove damaged protein and organelles and oppose apoptotic responses (Teng et al., 2011), and if it fails, cell apoptosis and tissue damage will occur. The apoptotic cells increased when the autophagy flux was blocked by chloroquine. A 2.0 g/cm^2^ static compressive force was used in this study, since previous study demonstrated that partial damage of PDLSCs occurred when the compressive force was at 4.0 g/cm^2^ (Kanzaki et al., 2002). There was no significant difference of apoptosis in the cells subjected to 2.0 g/cm^2^ static compression compared to the control cells; however, the apoptotic cells increased significantly when the autophagy was inhibited in the force group. Previous study showed that autophagy was sensitive to light compressive force (Chen et al., 2019); the heavy force-induced damage of PDLSCs may be due to the impaired autophagy response. The increased autophagy serves as a protective mechanism under compressive stress, and the investigation of autophagy would help to determine the optimum force during orthodontic tooth movement.

Further, we found lncRNA FER1L4 mediated the compressive force-induced autophagy in PDLSCs. LncRNA serves as essential regulators in diverse biological and pathological processes (Mattick, 2004). FER1L4 was one of the most highly upregulated transcripts in PDLSCs under static compressive force according to our previous study (Huang et al., 2019). Thus, we chose FER1L4 as the candidate lncRNA in the regulation of autophagy under mechanical stimulation. The data revealed that FER1L4 overexpression increased the autophagic cascades of PDLSCs, and knockdown of FER1L4 reversed the upregulated autophagy induced by compressive force. Previous studies have revealed the role of lncRNAs in regulating autophagy (Yang et al., 2017), further contributing to cancer development, inflammatory diseases, and cardiovascular disease, and thus lncRNAs are regard as potential therapeutic target of disease progression. Static compressive force is set to mimic the *in vivo* orthodontic treatment system. The upregulation of FER1L4 and the subsequent induction of autophagy may serve as a physiological reaction to protect the periodontal ligament tissue from damage under compressive force, and our findings may provide a promising target to improve orthodontic tooth movement.

FER1L4 regulated autophagic program via AKT/FOXO3 pathway. Mammalian target of rapamycin (mTOR), which is activated by PI3K/AKT signaling, is a major regulator of autophagy. FER1L4 inhibited the phosphorylation of AKT and thus inhibited the mTOR pathway to promote autophagy. Previous study indicated that FER1L4 inhibited AKT activation in carcinoma (Gao et al., 2019) by acting as miRNAs sponges to prevent miRNAs from binding their natural targets (Ye et al., 2019) or promoting the expression of phosphatase and tensin homolog (PTEN) (Qiao and Li, 2016; Sun et al., 2019), an antagonism of PI3K/AKT signaling pathway. However, lncRNA regulates gene expression by diverse mechanisms (Wang and Chang, 2011). The complex secondary structures enable them to interact with protein complexes, for example, lncRNA NKILA directly binds to p65 and inhibits NF-κB activation by masking the phosphorylation sites of IκB (Liu et al., 2015). The precise mechanism by which FER1L4 inhibited the phosphorylation of AKT needs further research. FOXO3, which belongs to the FOXO family, is a transcription factor that mediates the transcriptional activity of autophagy-related genes, including ATG7 (Yu et al., 2019), LC3, and Bnip3 (Cao et al., 2013). It is sufficient and necessary to initiate autophagy (Mammucari et al., 2007). AKT activation phosphorylates FOXO and blocks its nuclear translocation (Mammucari et al., 2007). Compressive strain reduced the phosphorylation of AKT and increased the nuclear translocation of FOXO3. In addition, FER1L4 inhibited AKT activation and subsequently increased FOXO3 activity, while knockdown of FER1L4 abolished the upregulation of FOXO3 induced by compressive stress, suggesting FER1L4 regulated compression-induced autophagy via AKT/FOXO3 pathway. However, lncRNA participates in autophagic program via various autophagy-related genes at the transcriptional, post-transcriptional, and translational levels, and other molecules may also be involved.

## Conclusion

Autophagy of periodontal ligament stem cells was induced by the orthodontic compressive force, and lncRNA FER1L4 mediated the activation of autophagy under compressive force via AKT/FOXO3 pathway (Figure 6D). These findings suggest a mechanism of autophagy regulation by lncRNA during periodontal tissue remodeling of orthodontic tooth movement.

## Data Availability Statement

The datasets presented in this study can be found in online repositories. The names of the repository/repositories and accession number(s) can be found in the article/supplementary material.

## Ethics Statement

The studies involving human participants were reviewed and approved by the Research Ethics Committee, Peking University School and Hospital of Stomatology (PKUSSIRB-2011007). Written informed consent to participate in this study was provided by the participants’ legal guardian/next of kin. The animal study was reviewed and approved by Institutional Animal Care and Use Committee, Peking University (LA2018305).

## Author Contributions

YHu performed the experiments, analyzed the data, prepared the figures and tables, and wrote the manuscript. HL assisted in the micro-CT and histologic assay. RG assisted in the data collection and analyses. YHa contributed to the FISH staining. YY contributed to the mouse orthodontic tooth movement experiments. YiZ assisted in the application of static compressive force. YuZ assisted in RNA sequencing data analyses. LJ edited the manuscript. WL supervised the work and edited the manuscript. All authors approved the final version of the manuscript.

## Conflict of Interest

The authors declare that the research was conducted in the absence of any commercial or financial relationships that could be construed as a potential conflict of interest.
